# Dark Triad Traits, Sex, and Social Desirability as Predictors of Non-Consensual Intimate Media Sharing Proclivity, Enjoyment, and Approval in UK University Students

**DOI:** 10.3390/bs15060781

**Published:** 2025-06-05

**Authors:** Charlotte Kite, Anthony Murphy, Melissa F. Colloff

**Affiliations:** School of Psychology, The University of Birmingham, Birmingham B15 2TT, UK; cxk273@student.bham.ac.uk (C.K.);

**Keywords:** non-consensual intimate media sharing, revenge pornography, dark triad traits, Machiavellianism, psychopathy, narcissism

## Abstract

Non-consensual intimate media sharing (NCIMS)—defined as the non-consensual sharing of sexually explicit images or videos—has notably increased in recent years, despite legislative actions to tackle this. This study aimed to investigate whether the Dark Triad traits of narcissism, Machiavellianism, and psychopathy—as well as sex and social desirability—predicted NCIMS proclivity, enjoyment, and approval in UK university students. A total of 653 participants were recruited through Prolific, the University of Birmingham survey circle, and social media. All participants completed various measures to assess self-reported levels of Dark Triad traits, social desirability, and NCIMS proclivity, enjoyment, and approval. The results showed that the models for each multiple linear regression (NCIMS proclivity, enjoyment, and approval) were statistically significant, though only certain variables were independent predictors for each regression. For proclivity, only psychopathy independently added to the prediction. For enjoyment, significant predictors were sex, Machiavellianism, and psychopathy. For approval, only Machiavellianism added statistically to the prediction. This research adds to the growing literature base around NCIMS, specifically within university students in the UK, and provides strong evidence for the development and implementation of interventions designed to address the likelihood of individuals perpetrating NCIMS.

## 1. Introduction

Non-consensual intimate media sharing (NCIMS)—colloquially known as “revenge pornography”—refers to the sharing of private sexual materials (photos or videos) of another person without their consent (Ministry of Justice, 2015). The term revenge pornography has been widely criticised for the negative connotations associated with it and for inferring that the victim must have done something to warrant the behaviour (i.e., in revenge) which has victim-blaming implications (Henry et al., 2020; McGlynn & Rackley, 2017). There are many terms within the literature to evidence this phenomenon, including—but not limited to—intimate image abuse, the non-consensual dissemination of intimate media, the non-consensual sharing of intimate images, technology-facilitated sexual abuse, and image-based sexual abuse. Across the literature, papers will have varying scopes for what behaviours specifically fall under these titles. For the purpose of this paper, the term NCIMS will be used specifically to encompass behaviours in which the perpetrator disseminated intimate media without the consent of the person depicted in it. This is to exclude other forms of image-based sexual abuse which are more contact-based in nature such as upskirting or downblousing, as well as manufactured intimate media known as “deep fakes”. Recently updated legal definitions in England and Wales (now formulated in Section 66B of the Sexual Offences Act; CPS, 2024; Online Safety Act, 2023) remove the burden of proving that the perpetrator intended to cause distress or embarrassment. Although this represents a positive legislative shift, charities such as the Revenge Porn Helpline (2024) caution that challenges remain in prosecuting and convicting perpetrators, including difficulties in establishing non-consent from content alone. These new laws now feature in Section 66B of the Sexual Offences Act, with the following legislative definition: “A person (A) commits an offence if: (A) intentionally shares a photograph or film which shows, or appears to show, another person (B) in an intimate state; (B) does not consent to the sharing of the photograph or film, and; (A) does not reasonably believe that (B) consents”. As this legislation change is relatively recent (January 2024), the majority of the literature citing NCIMS would be based upon the previous legal definition unless stated otherwise.

The noted legislative changes structure the NCIMS definition in line with that of rape or other forms of sexual assault within the Sexual Offences Act (2003), which could be considered to evidence the gradual development of understanding regarding the seriousness of these offences and the parallels between technology-facilitated “non-contact” sexual offending. Despite this positive change, the definition has still been subjected to caution around implementation—specifically, the concern that the definition of “consent” would be difficult to ascertain from the content alone (McGlynn et al., 2024). UK charity and support organisation Revenge Porn Helpline praised the legislative change, highlighting that the shift will allow for enhanced support for victims as well as begin a cultural shift in the severity of intimate image abuse (Revenge Porn Helpline, 2024).

Research demonstrates that NCIMS is increasing in prevalence (Slater & Gordon, 2021). The UK-based Revenge Porn Helpline reported an 87% increase in cases between 2019 and 2020 (Revenge Porn Helpline, 2021) and a further 40% increase in 2021 (Revenge Porn Helpline, 2022). The majority of victims are female, whereas men are more frequently the perpetrators (Branch et al., 2017; Hall & Hearn, 2017). Psychological impacts on victims commonly include depression, anxiety, trust issues, and post-traumatic stress symptoms (Bates, 2016), akin to the harm experienced by victims of contact sexual abuse (McGlynn et al., 2017). These harmful outcomes underscore the seriousness of NCIMS and highlight the importance of understanding factors that may predispose individuals to perpetrating it. As with other forms of sexual offending, it appears that NCIMS is a gendered crime. A large-scale study conducted across Australia, New Zealand, and the UK surveyed 6109 individuals regarding self-reported image-based sexual abuse behaviours (Powell et al., 2022). Powell et al. (2022) found that male participants were more likely to report engaging in IBSA perpetration collectively and when considering the separate forms (taking, sharing, and threatening to share). These findings are in line with other research that also identified that males are more likely to self-report perpetration than females (Powell et al., 2019). Additionally, research has also found that males have demonstrated more victim-blaming views towards victims of NCIMS than females do (Zvi & Bitton, 2021).

University students may be at particular risk. Branch et al. (2017) found that 10% of opportunistically surveyed students reported experiences with NCIMS as victims, and 62.1% felt social pressure from peers to engage in intimate media sharing. Similarly, Jaiswal (2021) found that of 100 college students, 12 had reported experiencing NCIMS or knowing someone who did. Friendship networks in university settings often replicate the intense peer bonds seen in adolescence (Buote et al., 2007; Richey & Richey, 1980), potentially contributing to normative or pressured behaviours around sexting.

University communities have also been scrutinised for “rape culture”, with student-focused organisations reporting higher rates of sexual misconduct compared to national averages (NUS, 2010, 2019; Office for National Statistics, 2017). Although research specific to UK university students and NCIMS remains limited, Gavin and Scott (2019) identified that a majority of students (55.2%) tend to attribute at least partial responsibility to victims for intimate images being leaked, reflecting an emerging narrative of trivialisation or normalisation of NCIMS on campuses.

The Dark Triad of personality comprises the traits Machiavellianism, narcissism, and psychopathy (Paulhus & Williams, 2002). A recent systematic review and meta-analysis identified that both psychopathy and narcissism were negatively associated with affective empathy and that Machiavellianism was negatively correlated with both cognitive and affective empathy (Shukla & Upadhyay, 2025). Each trait is implicated in interpersonal exploitation and reduced empathy, which may predispose certain individuals to sexual aggression (Jonason et al., 2017; O’Connell & Marcus, 2016; Wheeler et al., 2002). Indeed, Pina et al. (2017, 2021) found that Dark Triad traits correlated with an increased likelihood to distribute intimate images non-consensually, with psychopathy most strongly linked to NCIMS proclivity and Machiavellianism also predictive of image-based sexual abuse (IBSA). Moreover, narcissism was associated with heightened excitement and amusement regarding IBSA, while moral disengagement emerged as another relevant factor.

In addition, bias introduced by social desirability may shape self-report data about potentially illegal or stigmatised behaviours like NCIMS (Lavrakas, 2008). Pina et al. (2017) explicitly recommended addressing social desirability by using appropriate measures (e.g., the Crowne and Marlowe Social Desirability Scale (Crowne & Marlowe, 1960)) to clarify how self-presentation might obscure genuine willingness to engage in image-based abuse.

The current study aimed to investigate whether the Dark Triad traits (narcissism, Machiavellianism, and psychopathy), sex, and social desirability predict NCIMS proclivity, enjoyment, and approval among UK university students. Such research can extend our understanding of how particular personality profiles may heighten the risk of engaging in NCIMS behaviours on campus, thereby informing targeted psychoeducational or bystander interventions. The incorporation of a measure of social desirability would serve several purposes within this study: examine the social desirability of the participant sample, give insight into the extent to which the participants may be influenced by the atmosphere around them, and therefore give potential insight into the possibility of a rape culture within UK universities. High social desirability predicting NCIMS proclivity could lend evidence to support the conclusion that there is an environment within which the response to potentially engaging in NCIMS would be socially desirable.

Based on the extant literature, five hypotheses were tested. This study was conducted on the basis of the following hypotheses: that high levels of narcissism, Machiavellianism, psychopathy, and social desirability will predict NCIMS proclivity, enjoyment, and approval in participants. The study also hypothesised that male participants will score higher on NCIMS proclivity, enjoyment, and approval than female participants.

## 2. Methodology

### 2.1. Participants and Procedure

Participants were UK university students aged 18 or older (N = 653; 21.75% male, 78.25% female; mean = 23.36 years; SD = 8.19 years; range = 18–69). Recruitment was conducted via convenience sampling within and external to the University, via the University of Birmingham Research Participation Scheme, Prolific, social media, and posters around the university. Participants through Prolific were financially compensated at the recommended rate of GBP 6 per hour, participants via the Research Participation Scheme were compensated through credits, and participants through other media were not compensated. This inequality of remuneration was clearly stated on the participant briefing forms, giving all participants the informed right to consent with this knowledge. Individuals identifying as non-UK students or under 18 years of age were screened out post-data collection. A priori power analysis using G*Power (Faul et al., 2007) suggested that at least 647 participants were required to detect small effects in a multiple regression, based upon an effect size of 0.02 alongside an alpha value of 0.05 to achieve 80% power. This analysis was based upon the existing literature in the area reporting small sample sizes (Clancy et al., 2019; Muris et al., 2017; Timmermans et al., 2018). The final sample consisted of 653 valid responses after removing incomplete cases or individuals failing to meet eligibility criteria.

### 2.2. Design

A cross-sectional, correlational design was employed. Participants accessed the online Qualtrics survey through a link or QR code. After reading the participant information sheet and giving informed consent, individuals proceeded to complete demographic items (age, sex, ethnicity, university level), followed by the three main questionnaires (Dark Triad, social desirability, and Revenge Porn Proclivity Scale). The term “sharing of intimate media” was used in participant-facing materials to mitigate negative connotations and potential re-traumatisation.

Ethical approval was granted by the University of Birmingham Ethics Committee. All data were collected anonymously; participants were asked to create a unique ID for the possibility of withdrawal within 14 days of participation. After this period, data were integrated into the dataset and no longer traceable to individual identifiers.

### 2.3. Materials

#### 2.3.1. Dark Triad Traits

The Short Dark Triad (SD3; Jones & Paulhus, 2013) is a 27-item measure assessing Machiavellianism, narcissism, and psychopathy. Participants rated statements (e.g., “It’s not wise to tell your secrets”) on a 5-point Likert scale (1 = strongly disagree to 5 = strongly agree). Nine items are each loaded onto one of the three subscales (Machiavellianism, narcissism, psychopathy). Higher scores indicate higher levels of each trait, and there is a minimum possible score of 27 and a maximum possible score of 135. Internal reliabilities in this sample were acceptable to good (α = 0.75 for Machiavellianism, 0.74 for narcissism, and 0.72 for psychopathy), compared to original internal reliabilities as follows: α = 0.77 for Machiavellianism, α = 0.71 for narcissism, and α = 0.80 for psychopathy.

#### 2.3.2. Social Desirability

A 13-item short form of the Crowne–Marlowe Social Desirability Scale (Reynolds, 1982) was used to assess the tendency to answer in a socially approved manner. True/false responses receive a score of 1 if they match the social desirability key and 0 if not. Scores range from 0 to 13, with higher totals indicating higher social desirability. The scale’s alpha was 0.65 in this sample (originally 0.76; Reynolds, 1982).

#### 2.3.3. Non-Consensual Intimate Media Sharing (NCIMS) Proclivity, Enjoyment, and Approval

The Revenge Porn Proclivity Scale (RPPS; Pina et al., 2017) consists of five scenarios describing the non-consensual sharing of intimate images for “revenge” or retaliation motives. Each scenario is followed by a question about the likelihood of engaging in the same behaviour (scored on a 5-point scale, 1 = definitely would not do the same to 5 = definitely would do the same), generating a proclivity subscale score (possible range 5–25). Additional items measure emotional reactions to each scenario (excitement, control, amusement, blame, anger, and regret) grouped into two subscales: enjoyment (excitement, control, amusement) and approval (blame, anger, regret reversed). Higher sums reflect more enjoyment or approval of the depicted NCIMS behaviours. Cronbach’s α for each subscale in this sample was 0.85 (proclivity), 0.87 (enjoyment), and 0.77 (approval), compared to original internal reliabilities as follows: α = 0.76 for proclivity, α = 0.87 for enjoyment, and α = 0.80 for approval.

### 2.4. Analysis

Descriptive statistics were computed to describe the sample, followed by statistics analysing the endorsement of NCIMS, specifically the percentage of the sample which showed endorsement of NCIMS proclivity, enjoyment, and approval. Three multiple linear regressions and their assumptions (linearity, independence of residuals, normality, multicollinearity, and homoscedasticity) were calculated, one for each dependent variable: NCIMS proclivity, NCIMS enjoyment, and NCIMS approval. All three multiple linear regressions included the independent variables of Machiavellianism, narcissism, psychopathy, sex, and social desirability. All assumptions were met. The Bonferroni correction was applied throughout to account for type 1 error rates. The internal consistency reliability of all measures specific to this sample was also calculated.

## 3. Results

### 3.1. Scale Reliabilities and Descriptive Statistics

For the SD3, Cronbach’s α ranged from 0.72 (psychopathy) to 0.75 (Machiavellianism) and 0.74 (narcissism), closely approximating prior psychometric reports. The SD3 total α was 0.85. Social desirability showed α = 0.65, slightly lower than the originally reported 0.70 (Reynolds, 1982). For the RPPS subscales, α was 0.85 (proclivity), 0.87 (enjoyment), and 0.77 (approval), consistent with prior findings (Pina et al., 2017). See Table 1 for descriptive statistics. 

### 3.2. Endorsement of Revenge Porn

Following Pina et al. (2017), scores above the minimum on each subscale were considered an indication of some endorsement of that dimension. For proclivity, the minimum possible score is 5 (i.e., scoring “1” on each of the five items). In this sample, 23.1% (n = 151) reported scores above this minimal threshold, indicating at least some NCIMS proclivity. Enjoyment and approval were similarly examined: 86.7% endorsed some NCIMS enjoyment, while 99.2% endorsed some level of approval. See Table 2 for the sample endorsement of NCIMS. 

### 3.3. Multiple Linear Regression Analyses

Three standard multiple linear regressions were conducted to predict (1) NCIMS proclivity, (2) NCIMS enjoyment, and (3) NCIMS approval from Machiavellianism, narcissism, psychopathy, social desirability, and sex. Bonferroni corrections were applied to account for multiple comparisons, resulting in a more conservative alpha value of 0.016.

### 3.4. NCIMS Proclivity

The overall model was significant, with F(5, 647) = 16.05 and *p* < 0.001, with an adjusted *R*^2^ = 0.103, explaining 11% of variance, indicating a small effect size (Cohen, 2013). Only psychopathy contributed uniquely to the prediction (β = 0.27, *p* < 0.001). Neither Machiavellianism, narcissism, sex, nor social desirability reached significance in the final model. See Table 3 for results of the multiple linear regression for NCIMS proclivity. 

### 3.5. NCIMS Enjoyment

The second regression was significant, F(5, 647) = 19.24, *p* < 0.001, adjusted *R*^2^ = 0.123, explaining 12.9% of shared variance, again reflecting a small effect size (Cohen, 2013). Psychopathy (β = 0.17, *p* < 0.001), Machiavellianism (β = 0.15, *p* < 0.001), and being male (β = 0.08, *p* = 0.038) emerged as significant predictors of enjoyment. Narcissism and social desirability did not predict unique variance. See Table 4 for results of the multiple linear regression for NCIMS enjoyment.

### 3.6. NCIMS Approval

The final model was also significant, with F(5, 647) = 14.05, *p* < 0.001, and adjusted *R*^2^ = 0.091, explaining 9.8% of shared variance, again reflecting a small effect size (Cohen, 2013). Machiavellianism (β = 0.22, *p* < 0.001) was the only significant predictor, whereas narcissism, psychopathy, social desirability, and sex did not uniquely contribute. See Table 5 for results of the multiple linear regression for NCIMS approval. 

## 4. Discussion

This work tested whether the Dark Triad, sex, and social desirability tendencies clarify three facets of non-consensual intimate media sharing (NCIMS): proclivity, affective reward, and moral approval. Although the statistical models accounted for only a small share of variance, the pattern of predictors enriches present knowledge in several ways and offers a baseline against which the January 2024 reforms that inserted Section 66B into the Sexual Offences Act can later be evaluated.

Psychopathy alone raised the odds of both willingness and anticipated enjoyment. This finding reproduces earlier UK work that linked callous impulsivity to image-based abuse (Pina et al., 2017, 2021) and accords with broader evidence of limited empathic concern among individuals high on this trait (Jonason et al., 2013). In line with O’Connell and Marcus (2016), psychopathy here fuelled excitement rather than explicit justification; once enjoyment entered the equation, approval no longer tracked psychopathy. This pattern hints that some perpetrators act for thrill without construing their conduct as legitimate, reinforcing the value of separating affective and cognitive components when studying technology-facilitated sexual aggression.

Machiavellianism predicted moral approval and shared variance in enjoyment, suggesting a colder, more strategic pathway. Prior observations that Machiavellian individuals neutralise victim harm to secure instrumental goals (Pina et al., 2021) gain support: participants high on this trait were more likely to regard NCIMS as defensible, even when psychopathy and sex were controlled. Their stance mirrors moral-disengagement scripts common in online harassment and aligns with survey work indicating that planned dissemination often serves reputational leverage (Clancy et al., 2019).

Men reported higher enjoyment, yet sex carried no unique weight for proclivity or approval. Campus qualitative studies show that male peer groups sometimes celebrate image circulation as humorous or status-enhancing (Branch et al., 2017; Hall & Hearn, 2017). The present data suggest that this peer reinforcement inflates anticipated pleasure rather than altering conscious moral stance. Because only one-fifth of the sample were male, statistical power to detect additional sex effects was restricted; future quotas or oversampling is therefore necessary before firm conclusions on gendered risk can be drawn.

More than four-fifths of respondents expressed at least some enjoyment, and almost the entire sample indicated at least minimal approval. Whilst these findings do not conclusively suggest that these participants expressed complete approval of NCIMS or would enjoy NCIMS content explicitly, by expressing a level above the minimum, it would suggest that there is some acceptance of this behaviour. Three contextual threads may explain these remarkable figures. First, UK student unions continue to document “lad” subcultures that trivialise sexual misconduct (NUS, 2019). Second, just-world reasoning, wherein victims are blamed for their misfortune, remains widespread in NCIMS contexts (Lerner & Miller, 1978; Attrill-Smith et al., 2021). Third, data collection preceded the Crown Prosecution Service guidance that publicised the new offence (CPS, 2024); limited legal awareness is known to relax perceived deterrence (Starr & Lavis, 2018).

### 4.1. Implications

The current results emphasise the potential utility of targeted psychoeducation or bystander interventions in reducing NCIMS perpetration on university campuses. Given the connections between Dark Triad traits and reduced empathy (Jonason et al., 2013), interventions emphasising victim impact may have limited effect on highly psychopathic or Machiavellian individuals. However, educational programmes detailing clear legal and personal consequences (e.g., criminal charges, reputational harm, academic sanctions) might engage those motivated to avoid self-risk. Bystander training that cultivates empathy and counters peer-group normalisation (Foubert & Perry, 2007) could also prove beneficial, as would the provision of information regarding clear reporting pathways to support victims in seeking support and reporting NCIMS behaviours. Interventions may also provide the opportunity to dispel myths that NCIMS is not as damaging to victims as physical sexual abuse, as supported by the literature (McGlynn et al., 2017). The combination of psychoeducation regarding legal consequences, increasing empathy for potential victims through victim-impact content, informative reporting pathways, and bystander intervention principles will likely prove successful in reducing what may be commonly held NCIMS-specific “rape myths”, therefore hopefully making pro-NCIMS views less desirable. Implementation during “freshers’ week” and on an ongoing basis throughout the academic year may help shift normative campus cultures around sexual consent and technology-facilitated abuse. This research has also further contributed to the literature base around NCIMS perpetration specifically within UK university students and has further validated the scales within this population with good internal consistencies—the Short Dark Triad (α = 0.85) and Revenge Porn Proclivity Scale respective subscales of proclivity (α = 0.85), enjoyment (α = 0.87), and approval (α = 0.77).

Earlier investigations with smaller samples linked Dark Triad traits to a selection of NCIMS indices (Pina et al., 2017, 2021). By modelling proclivity, enjoyment, and approval concurrently, and by adding an explicit social desirability check (Lavrakas, 2008), the present work reveals divergent psychological routes and confirms that impression management does not wholly account for self-reported willingness. The sample of more than six hundred students further improves parameter stability and establishes a pre-legislative benchmark that future longitudinal research can use when gauging cultural impact.

The dual-pathway picture indicates that impulsive offenders, driven by excitement, may respond to swift platform-level removal and visible sanctions, whereas strategic offenders require interventions that dismantle justificatory narratives and emphasise professional and legal costs. Bystander schemes that challenge masculine peer humour have already reduced sexual assault tolerance on campuses (Foubert & Perry, 2007) and could be adapted to NCIMS. Universities should pair such programmes with regular briefings on Section 66B so that ignorance of liability can no longer temper perceived risk.

### 4.2. Limitations

Several limitations should be considered when interpreting these findings. First, all data were self-reported and vulnerable to inaccuracies due to stigma or dishonest responding, even with anonymity procedures in place (Lavrakas, 2008). Second, the internal consistency of the Short Social Desirability measure (Reynolds, 1982) was found to be lower in this sample at *α* = 0.65 than would be expected, given the original scale development of 0.76. Third, the reliance on convenience sampling yielded a sample predominantly of female participants, limiting the potential generalisability of findings related to sex differences. However, a post hoc sensitivity analysis in G*Power shows that, with n_1_ = 142 (men) and n_2_ = 511 (women), this study retains 80% power (α = 0.05, two-tailed) to detect a Cohen’s d of ≈0.25—the magnitude reported in the most recent multi-country meta-analysis of image-based sexual-abuse attitudes (Powell et al., 2022). It is likely that if the sample was more balanced across males and females, based on significant research in the area suggesting that NCIMS and IBSA are gendered crimes (Branch et al., 2017; Hall & Hearn, 2017; Powell et al., 2019, 2022; Zvi & Bitton, 2021), sex would be a more significant predictor than seen in the current study. Conclusions regarding sex within this research should be considered in light of this limitation.

Future investigations should replicate these analyses with more balanced sex distributions to better elucidate gendered patterns in NCIMS attitudes and behaviours. Additionally, there is scope for designing and validating psychometric tools that measure dimensions such as victim blaming and rape myth acceptance specifically within NCIMS contexts. Interventions tailored to technology-facilitated sexual abuse could then be evaluated for efficacy using these targeted measures. An expanded focus on other forms of image-based sexual abuse (e.g., upskirting) specifically within the UK university student population would further refine our understanding of these behaviours and potentially inform broader policies to combat online sexual violence.

Similarly, the data for this study were collected prior to the new legislation that came into effect in January 2024, and thus, future research may benefit from evaluating the shift in the public perception of NCIMS. Of particular interest may be consideration of victim blaming in cases of NCIMS, which could be directly compared to prior findings suggesting that victim blaming is rife within this offence type (McKinlay & Lavis, 2020; Starr & Lavis, 2018). It is possible that the legislation change regarding the removal of the requirement to prove intent to cause distress or embarrassment will influence how victims of NCIMS are viewed.

## 5. Conclusions

Psychopathy, Machiavellianism and male sex explain distinct portions of variance across behavioural, affective, and cognitive responses to NCIMS. By demonstrating that thrill-seeking and instrumental motives operate side by side, this study advances theoretical models of image-based sexual abuse and supplies evidence for targeted counter-measures at precisely the moment that UK law begins to treat intimate-image offences with the seriousness they warrant.

## Figures and Tables

**Table 1:** Means (M) and standard deviations (SD) for each variable.

Variable	M	SD
Narcissism	23.0	5.36
Machiavellianism	27.4	5.25
Psychopathy	18.8	5.10
Social Desirability	6.43	2.64
NCIMS Proclivity	5.78	2.18
NCIMS Enjoyment	26.19	9.13
NCIMS Approval	37.36	8.84

**Table 2:** Sample endorsement of NCIMS.

	Proclivity	Enjoyment	Approval
*n*	151	566	648
%	23.13%	86.68%	99.23%

**Table 3:** MLR results for NCIMS proclivity.

NCIMS Proclivity	*B*	95% CI for *B*		*SE B*	β	*t*	*p*	*R* ^2^	Adj. *R*^2^
		*LL*	*UL*						
Model								0.110	0.103
(Constant)	3.04	1.85	4.24	0.61		4.99	<0.001		
Sex	0.37	−0.03	0.78	0.21	0.07	1.82	0.07 ^t^		
Machiavellianism	0.000	−0.04	0.04	0.02	0.001	0.02	0.99		
Narcissism	0.02	−0.01	0.06	0.02	0.06	1.38	0.17		
Psychopathy	0.12 *	0.08	0.16	0.02	0.27	5.65	<0.001		
Social desirability	−0.01	−0.08	0.06	0.03	−0.01	−0.33	0.74		

*Note.* Model = “Enter” method in SPSS Statistics Version 29; *B* = unstandardised regression coefficient; CI = confidence interval; *LL* = lower limit; *UL* = upper limit; *SE B* = standard error of the coefficient; β = standardised coefficient; *R*^2^ = coefficient of determination; Adj. *R*^2^ = adjusted *R*^2^. * = *p* < 0.001”. ^t^ = trending towards significance.

**Table 4:** MLR Results for NCIMS Enjoyment.

NCIMS Enjoyment	*B*	95% CI for *B*		*SE B*	β	*t*	*p*	*R* ^2^	Adj. *R*^2^
		*LL*	*UL*						
Model								0.129	0.123
(Constant)	10.67 *	5.7	15.64	2.53		4.22	<0.001		
Sex	1.78 **	0.1	3.45	0.85	0.08	2.08	0.04		
Machiavellianism	0.27 *	0.12	0.42	0.08	0.15	3.46	<0.001		
Narcissism	0.11	−0.03	0.25	0.07	0.06	1.53	0.13		
Psychopathy	0.31 *	0.15	0.48	0.09	0.17	3.68	<0.001		
Social desirability	−0.09	−0.36	0.19	0.14	−0.03	−0.63	0.53		

*Note.* Model = “Enter” method in SPSS Statistics Version 29; *B* = unstandardised regression coefficient; CI = confidence interval; *LL* = lower limit; *UL* = upper limit; *SE B* = standard error of the coefficient; β = standardised coefficient; *R*^2^ = coefficient of determination; Adj. *R*^2^ = adjusted *R*^2^. * = *p* < 0.001 ** = *p* < 0.05.

**Table 5:** MLR results for NCIMS approval.

NCIMS Approval	*B*	95% CI for *B*		*SE B*	β	*t*	*p*	*R* ^2^	Adj. *R*^2^
		*LL*	*UL*						
Model								0.098	0.091
(Constant)	22.49 *	17.59	27.38	2.49		9.02	<0.001		
Sex	0.09	−1.56	1.74	0.84	0.004	0.1	0.92		
Machiavellianism	0.37 *	0.22	0.52	0.08	0.22	4.91	<0.001		
Narcissism	0.19	−0.02	0.26	0.07	0.07	1.65	0.1		
Psychopathy	0.13	−0.04	0.29	0.08	0.07	1.52	0.13		
Social desirability	−0.07	−0.35	0.2	0.14	−0.02	−0.53	0.59		

*Note.* Model = “Enter” method in SPSS Statistics Version 29; *B* = unstandardised regression coefficient; CI = confidence interval; *LL* = lower limit; *UL* = upper limit; *SE B* = standard error of the coefficient; β = standardised coefficient; *R*^2^ = coefficient of determination; Adj. *R*^2^ = adjusted *R*^2^. * = *p* < 0.001.

## Data Availability

The original contributions presented in the study are included in the article, further inquiries can be directed to the corresponding author.
